# Thanatin: A Promising Antimicrobial Peptide Targeting the Achilles’ Heel of Multidrug-Resistant Bacteria

**DOI:** 10.3390/ijms25179496

**Published:** 2024-08-31

**Authors:** Qianhui Liu, Qian Wu, Tianming Xu, Pradeep K. Malakar, Yongheng Zhu, Jing Liu, Yong Zhao, Zhaohuan Zhang

**Affiliations:** 1 College of Food Science and Technology, Shanghai Ocean University, 999# Hu Cheng Huan Road, Shanghai 201306, China; m220351069@st.shou.edu.cn (Q.L.); d220300083@st.shou.edu.cn (Q.W.); d230300096@st.shou.edu.cn (T.X.); pkmalakar@shou.edu.cn (P.K.M.); yh-zhu@shou.edu.cn (Y.Z.); d210300079@st.shou.edu.cn (J.L.); 2 International Research Center for Food and Health, Shanghai Ocean University, 999# Hu Cheng Huan Road, Shanghai 201306, China

**Keywords:** thanatin, Achilles’ heel, multidrug-resistant bacteria

## Abstract

Antimicrobial resistance poses an escalating threat to human health, necessitating the development of novel antimicrobial agents capable of addressing challenges posed by antibiotic-resistant bacteria. Thanatin, a 21-amino acid β-hairpin insect antimicrobial peptide featuring a single disulfide bond, exhibits broad-spectrum antibacterial activity, particularly effective against multidrug-resistant strains. The outer membrane biosynthesis system is recognized as a critical vulnerability in antibiotic-resistant bacteria, which thanatin targets to exert its antimicrobial effects. This peptide holds significant promise for diverse applications. This review begins with an examination of the structure–activity relationship and synthesis methods of thanatin. Subsequently, it explores thanatin’s antimicrobial activity, detailing its various mechanisms of action. Finally, it discusses prospective clinical, environmental, food, and agricultural applications of thanatin, offering valuable insights for future research endeavors.

## 1. Introduction

In recent years, there has been a notable increase in resistance among pathogenic microorganisms towards conventional antimicrobial agents [1]. This trend underscores antimicrobial resistance as a pressing global public health concern [2], posing a significant threat to human health on a worldwide scale [3,4]. In 2019 alone, six predominant resistant pathogens, namely, *Escherichia coli*, *Staphylococcus aureus*, *Klebsiella pneumoniae*, *Streptococcus pneumoniae*, *Acinetobacter baumannii*, and *Pseudomonas aeruginosa*, were directly responsible for 1.27 million deaths and contributed indirectly to an additional 4.95 million deaths [5]. According to the World Health Organization, if left unchecked, antimicrobial resistance could lead to an astonishing $100 trillion loss to the global economy by 2050 [6]. Despite these dire projections, progress in developing effective drugs against resistant bacteria has been slow in recent decades, emphasizing the urgent need for novel antimicrobial strategies.

Thanatin, a β-hairpin cationic peptide featuring a single disulfide bond, was initially isolated from the hemiptera insect *Podisus maculiventris* in 1996 and demonstrates broad-spectrum antimicrobial activity [7,8,9]. The outer membrane (OM) component biosynthesis system, encompassing lipopolysaccharide transport (Lpt), maintenance of lipid asymmetry (Mla), β-barrel assembly machinery (Bam), and localization of lipoprotein (Lol), represents critical vulnerabilities in antibiotic-resistant bacteria [10,11]. Common secondary structures include α-helices and β-hairpins [12]. Compared to α-helical structures, β-hairpin structures are generally more stable and less compressible [13]. As a β-hairpin cationic peptide, thanatin selectively targets these vulnerabilities, effectively combating resistant strains. Thus, in the context of severe antimicrobial resistance, thanatin emerges as a promising peptide with extensive potential applications.

This review begins by examining the structure–activity relationship of thanatin and detailing its synthesis methods. Subsequently, it explores the antimicrobial activity of thanatin, elucidating its diverse mechanisms of action. Finally, it discusses the multifaceted potential applications of thanatin as an antimicrobial agent. The insights presented herein aim to provide valuable references for future research endeavors focused on thanatin.

## 2. Structure–Activity Analysis of Thanatin

**Figure 1:**
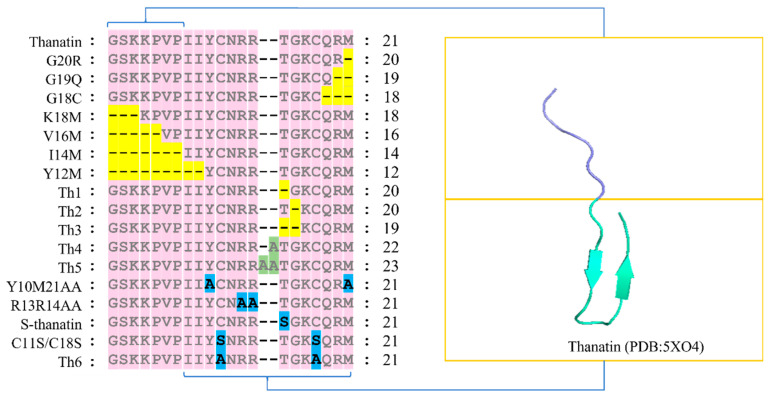
The analogs of thanatin are obtained through the deletion, addition, and replacement of amino acids. In the representation, pink indicates the original amino acids of thanatin; yellow indicates the deleted amino acids; green indicates the added amino acids; and blue indicates the replaced amino acids.

Thanatin is a small molecular antimicrobial peptide composed of 21 amino acids (GSKKPVPIIYCNRRTGKCQRM) [7]. Its structure comprises two distinct segments: (1) the first 7 N-terminal amino acids (G1 to P7) primarily consist of hydrophobic residues, and (2) the subsequent 14 amino acids (I8 to M21) adopt an antiparallel β-fold, stabilized by a disulfide bond between Cys 11 and Cys 18. Modifications in the peptide sequence, such as deletion, addition, or substitution of amino acids, have been shown to influence its antimicrobial activity [8,14,15]. This review outlines the sequence of analogs derived from these modifications to thanatin (Figure 1) and evaluates their impact on its antimicrobial efficacy.

### 2.1. Relationship between Residues G^1^-P^7^ and the Antimicrobial Activity of Thanatin 

The amino acid sequence G^1^SKKPVP^7^of thanatin adopts a conformation characterized by mixed elements, including extension and a β-turn. Specifically, residues K^3^KPV^6^ form a β-turn centered on K4 and P5. This β-turn conformation likely facilitates interaction between the cationic side chains of K3 and K4 with the cationic side chain of R20 at the N-terminus of the β-chain [16]. The N-terminal segment G^1^SK^3^ plays a critical role in stabilizing the inverted parallel β-folding structure and is essential for its antifungal activity. For instance, the deletion of this segment diminishes the antimicrobial efficacy of the K18M analog against *Neurospora crassa* [17]. Moreover, the analog V16M, resulting from the absence of the N-terminal G^1^SKKP^5^ amino acids, exhibits reduced antimicrobial activity against bacteria such as *K. pneumoniae* [8]. Furthermore, complete deletion of all seven amino acids G^1^SKKPVP^7^ from the N-terminus further diminishes the antimicrobial potency of the I14M analog against *K. pneumoniae* [8].

### 2.2. Relationship between Residues I^8^-M^21^ and the Antimicrobial Activity of Thanatin

The amino acid sequence I^8^IYCNRRTGKCQRM^21^ of thanatin adopts a canonical β-hairpin conformation [7], a hallmark structural motif observed in both aqueous and detergent environments. Thanatin is known for its antimicrobial activity [16,18], which is underpinned by the formation of crucial disulfide bonds involving Cys11 and Cys18 [7,8]. Deletion of residues G^1^-G^9^, resulting in the Y12M analog, abolishes its antimicrobial efficacy against Gram-negative bacteria and fungi, while demonstrating inhibitory effects against specific Gram-positive bacteria such as *Aerococcus viridans*, *Micrococcus luteus*, and *Bacillus megaterium*, with minimum inhibitory concentrations (MICs) ranging from 20 to 40 µM [8].

The fifteenth residue, Thr, serves as a focal point in the disulfide ring, segregating positively charged residues into distinct subgroups [8]. Analogs Th1, Th2, and Th3, derived from deletions at T15, G16, or both T15 and G16, exhibit enhanced inhibitory effects against Gram-positive bacteria [14]. Analogs Th4, Th5, and R10M21AA show reduced antimicrobial activity, particularly against *Bacillus subtilis* and *S. aureus* [14,19]. The R13R14AA analog, with simultaneous mutations of R13 and R14 to Ala, displays virtually no bactericidal activity [16,19]. Alanine scanning is a site-directed mutagenesis technique [20,21] that systematically replaces amino acids in sequences with Ala to investigate the effects of various amino acids in thanatin on its antibacterial activity.

S-thanatin, where Thr is substituted with Ser [22,23], demonstrates heightened antimicrobial potency and reduced hemolytic toxicity compared to thanatin, particularly effective against Gram-negative bacteria such as *E. coli* [24]. However, replacing both C11 and C18 with Ser significantly diminishes the antimicrobial activity of the C11S/C18S analog against *E. coli*, while retaining inhibitory effects against *M. luteus*, suggesting differential mechanisms of action against these organisms [25]. Analogs with Ala substitutions for both Cys residues exhibit minimal antimicrobial activity [14], underscoring the essential role of disulfide bonds in antimicrobial efficacy.

The C-terminal amino acids Q^19^RM^21^ play a critical role in combating Gram-negative bacteria and certain Gram-positive strains. Analogs G18C and G19Q, lacking efficacy against *K. pneumoniae*, *Erwinia carotovora*, and *P. aeruginosa*, also show no inhibitory effect on *B. subtilis* [8].

The amino acid sequence I^8^IYCNRRTGKCQRM^21^ of thanatin adopts a canonical β-hairpin conformation [7], which is recognized as its characteristic structural motif, stable in both aqueous and detergent environments. Thanatin’s biological activity is attributed to this structural feature [16,18].

## 3. Synthetic Method of Thanatin

Methods employed for the production of thanatin encompass natural extraction, chemical synthesis, and genetic engineering. Owing to the sparse occurrence of thanatin in tissues and its limited natural abundance, extracting and purifying this antimicrobial peptide presents challenges, hindering the procurement of substantial quantities from biological sources. This review aims to outline prevalent techniques utilized in the synthesis of thanatin, including recombinant protein expression and solid-phase synthesis.

### 3.1. Recombinant Protein Expression

Recombinant expression of thanatin through genetic engineering represents an efficient approach for its production and purification. High-throughput recombinant expression systems utilized for thanatin include the *Pichia pastoris*, HEK293, and *E. coli* expression systems.

*P. pastoris* is recognized for its capability to effectively express recombinant proteins [26], offering notable advantages in terms of cost effectiveness and safety. Sabokkhiz et al. successfully produced thanatin in *P. pastoris* transformants harboring a pPICZαA vector containing the thanatin-coding sequence, resulting in secretion of the peptide into the growth medium. The produced thanatin demonstrated antiviral activity against the *tobacco mosaic virus* (TMV) [27].

Human embryonic kidney cells (HEK293) are prominent platforms for recombinant peptide production, known for their robust expression capabilities [28,29]. Studies have indicated higher expression levels of thanatin in HEK293 cells compared to *P. pastoris* [30]. Tanhaeian et al. engineered a recombinant plasmid for thanatin using pGH and pcDNA3.1+ vectors, transfected it into HEK293 cells, and successfully produced recombinant thanatin. The expressed peptide exhibited potent antimicrobial activity against *S. aureus* ATCC 12923 and *S. aureus* ATCC 25923, with the highest MIC value observed against *E. coli* ATCC 25922 [31]. Furthermore, Mamarabadi, Tanhaeian, and Ramezany constructed a recombinant plasmid, pcDNA™3.1(+)-thanatin, which enabled synthesis and secretion of recombinant thanatin in HEK293 cells. The produced thanatin demonstrated inhibitory effects against *Geotrichum candidum*, *Botrytis cinerea*, *Rhizoctonia solani*, *Alternaria tenuissima*, and *Gibberella fujikuroi* [32].

The *E. coli* expression system represents another widely used prokaryotic platform for producing thanatin, offering distinct advantages including a well-defined genetic background, straightforward operation, economical culture conditions, low production costs, and rapid growth [33,34]. Due to thanatin’s simple molecular structure, both its primary sequence and spatial conformation remain unchanged following expression in the *E. coli* system. Zhao et al. developed the S-thanatin-Trxa-pET32a expression vector incorporating a thrombin cleavage site and successfully expressed the S-thanatin fusion protein in *E. coli* BL21 (DE3). Following efficient cleavage of the S-thanatin fusion protein using immobilized thrombin, they purified the recombinant S-thanatin using preparative reverse-phase high-performance liquid chromatography. The purified recombinant exhibited potent biological activity against *E. coli* ATCC 25922 [35].

### 3.2. Solid-Phase Synthesis

Currently, the amino acid sequence of thanatin has been elucidated, and it can be synthesized using solid-phase peptide synthesis (SPPS) technology. Merrifield et al. pioneered solid-phase peptide synthesis in 1963, where the initial amino acid at the C-terminus of the target peptide is covalently bonded to a solid-phase support, with subsequent amino acids added sequentially from the N-terminus [36]. Solid-phase synthesis involves assembling amino acids in accordance with thanatin’s sequence template. Fmoc peptide synthesis, a derivative of SPPS, is widely preferred due to its mild reaction conditions, minimal side reactions, and high yields [36,37,38]. In Fmoc chemistry, the first amino acid is attached to resin; the peptide is elongated and then cleaved from the resin postsynthesis [39]. Vetterli et al. demonstrated that thanatin synthesized via Fmoc solid-phase synthesis exhibits potent activity against *E. coli* and its biofilms [40].

## 4. Antimicrobial Activity of Thanatin

Thanatin, an insect antimicrobial peptide, exhibits broad-spectrum antimicrobial properties effective against Gram-positive bacteria, Gram-negative bacteria, and filamentous fungi [8]. Notably, its antibacterial potency against Gram-negative bacteria surpasses that against Gram-positive bacteria and fungi [23,41]. Moreover, thanatin demonstrates efficacy against multidrug-resistant bacteria including *E. coli*, *K. aerogenes*, and *K. pneumoniae* [42]. Furthermore, derivatives of thanatin have shown enhanced antimicrobial activity.

Analogs of thanatin encompass S-thanatin [43], A-thanatin (C-amidated thanatin) [44], L-thanatin (linear thanatin) [45], C-thanatin (cyclic thanatin) [40], D-thanatin [8], and R-thanatin [46]. It is particularly noteworthy that D-thanatin shows no activity against Gram-negative bacteria, indicating a potential chiral target, while retaining efficacy against Gram-positive strains [40].

This review provides a comprehensive overview of the antimicrobial efficacy of thanatin, A-thanatin, and S-thanatin against both Gram-negative and Gram-positive bacteria. Furthermore, it examines the combined antimicrobial effects of thanatin with other agents. Variations in the minimum inhibitory concentration (MIC) of thanatin against different bacteria may be attributed to varying interactions between thanatin and bacterial proteins such as LptA and/or LptD [47]. Additionally, the antimicrobial activity of thanatin appears to be influenced by salt concentration, with MIC values increasing at higher NaCl concentrations [7,15].

### 4.1. Gram-Negative Bacteria

Table 1 presents the minimum inhibitory concentrations (MIC) of thanatin, A-thanatin, and S-thanatin against Gram-negative bacteria in vitro. Thanatin shows strong antimicrobial activity against various Gram-negative strains including *E. coli* [8], *Salmonella typhimurium* [8], *K. pneumoniae* [8], *Enterobacter cloacae* [8], *Salmonella enterica* [19], and NDM-1-producing *E. coli* and *K. pneumoniae* isolates [48], with MIC values below 3.2 µM. However, its efficacy against *Citrobacter freundii*, *Erwinia amylovora*, *Salmonella paratyphi* C, and *Shigella dysenteriae* is slightly reduced, with MIC values below 18 µM [17,31,49]. Sinha et al. observed potent inhibition of *P. aeruginosa* ATCC 27853 by thanatin, with an MIC of 1 µM [19]. Conversely, thanatin displays weaker activity against *E. carotovora*, with an MIC of 20 µM [8].

A-thanatin demonstrates potent bactericidal activity against multidrug-resistant *E. coli* (MDR-EC), multidrug-resistant *P. aeruginosa* (MDR-PA), extended-spectrum beta-lactamase-producing *E. coli* (ESBL-EC), extended-spectrum beta-lactamase-producing *K. pneumoniae* (ESBL-KP), and methicillin-resistant *Staphylococcus epidermidis*, with MIC values below 6.4 µM, highlighting its potential as a novel therapeutic strategy against pathogenic infections [44]. Compared to thanatin, A-thanatin shows similar efficacy against *E. coli*, *S. typhimurium*, *E. cloacae*, *E. carotovora*, and *K. pneumoniae* [44], but demonstrates superior antimicrobial activity against *P. aeruginosa*, with an MIC of 5 µM [8].

S-thanatin retains the antimicrobial potency observed in thanatin against *E. coli* and *K. pneumoniae* [24], with an MIC value of 5 µM recorded against *E. coli* E79466 [43]. Additionally, S-thanatin demonstrates significant antimicrobial effectiveness against *Klebsiella ornithinolytica* ATCC 31898, *Klebsiella oxytoca* ATCC 43086, *E. cloacae* ATCC 13047, and *Enterobacter aerogenes* ATCC 49701 [23]. However, compared to conventional therapy involving colistin, S-thanatin exhibits a less pronounced antimicrobial effect against *P. aeruginosa* [50]. Nonetheless, S-thanatin displays notable efficacy against multidrug-resistant strains of *K. pneumoniae*, particularly when used concomitantly with traditional antibiotics such as cefepime. This synergistic interaction is supported by an average fractional inhibitory concentration (FIC) of 0.3708 and shows a reduced potential for inducing resistance [51].

### 4.2. Gram-Positive Bacteria

Table 2 presents the minimum inhibitory concentration (MIC) values of thanatin, A-thanatin, and S-thanatin against Gram-positive bacteria. Thanatin demonstrates potent inhibitory activity against various Gram-positive bacteria, including *A. viridans*, *M. luteus*, *B. megaterium*, and *B. subtilis*, with MIC values below 5 µM. Particularly noteworthy is its strong antimicrobial effect against *Enterococcus faecalis* ATCC 29212 and *Streptococcus pyogenes* ATCC 19615, with MIC values of 0.5 µM [19]. However, both thanatin and A-thanatin show reduced sensitivity to *Pediococcus acidolactici* [8]. Sinha et al. reported a MIC value of 1 µM for thanatin against *S. aureus* ATCC 25923 [19]. Furthermore, Tanhaeian et al. observed that recombinant expression of thanatin exhibited potent antimicrobial activity against *S. aureus* ATCC 12923 but displayed a higher MIC value against *Listeria monocytogenes* [31]. Thanatin demonstrates inhibitory effects on methicillin-resistant *S. epidermidis* (MRSE), *Staphylococcus haemolyticus*, and *Staphylococcus hominis* at 3.2 µM, with slightly higher MIC values recorded for methicillin-resistant *S. aureus* (MRSA) ATCC 33591 and MRSA XJ75302, at 3.844 µM and 25.6 µM, respectively [44,45,46].

The antimicrobial activity of A-thanatin against Gram-positive bacteria such as *A. viridans* and *M. luteus* is comparable to that of thanatin. However, its inhibitory effect on *B. subtilis* and MRSE XJ75284 is relatively weaker, with MIC values of 5 µM [8] and 6.4 µM [44], respectively. Additionally, A-thanatin exhibits a high MIC value of 102.4 µM [44] against both *S. aureus* ATCC 29213 and MRSA XJ75302. The antimicrobial efficacy of S-thanatin against *B. subtilis* ATCC 21332 and *E. faecalis* ATCC 29212 is inferior to that of thanatin, with MIC values of 3.2 µM and 25.6 µM, respectively [15,23].

### 4.3. Antimicrobial Activity of Thanatin Combined with Other Antimicrobials

Thanatin employs two strategies to enhance its antimicrobial efficacy in combination with other antimicrobials. Firstly, it synergizes with other antimicrobial peptides, thereby augmenting their antimicrobial activity while reducing the necessary concentration of each peptide. This approach not only lowers costs but also achieves a better balance between effectiveness and potential toxicity. For instance, the combination of thanatin with cathelicidin-BF15-a3 demonstrates synergistic effects against *E. coli* O157:H7 (FICI = 0.375), exhibiting stronger antimicrobial efficacy at lower concentrations compared to individual treatments, which enhances its applicability in food safety [52].

Secondly, genetic engineering technology is utilized to design and synthesize novel antimicrobial peptides. Synthetic peptides offer a broad spectrum of antimicrobial activity, enhanced in vivo stability, increased biological activity, and lower cytotoxicity compared to natural peptides. Jiang et al. fused Melittin [53] with thanatin [54] to create the fusion peptide melittin-thanatin (MT), significantly boosting its antimicrobial activity against pathogens such as *S. aureus*, *B. subtilis*, and *S. typhi* [55]. Building upon MT, Liu et al. further modified the peptide by substituting three glycine (Gly, G) residues with tryptophan (Trp, W), resulting in MT-W, which exhibited improved inhibitory effects against *E. coli* K88 with reduced cytotoxicity and increased stability [56].

In another approach, Liu et al. designed the cecropinA-thanatin gene, which, upon expression and purification in the *Pichia pastoris* system, demonstrated potent antimicrobial activity against multidrug-resistant *A. baumannii* [30]. Wang et al. employed overlapping extension gene splicing technology to obtain the heterologous peptide gene attacin-thanatin, which exhibited slightly enhanced expression and demonstrated nontoxicity to porcine red blood cells while effectively inhibiting the growth of *E. coli* DH5α, *E. coli* BL21 (DE3), *Salmonella choleraesuis*, and *S. aureus* [57]. Additionally, Fan et al. developed an antimicrobial protein named TGH (thanatin-GFP-HBSAG), comprising thanatin, green fluorescent protein (GFP), and HBsAg (hepatitis B virus surface antigen). TGH binds to Gram-negative bacterial cells and integrates into lipid bilayers, facilitating accelerated clearance of multidrug-resistant E. coli in mice vaccinated with the TGH vaccine [58].

## 5. Multiple Bactericidal Mechanisms of Thanatin

**Figure 2:**
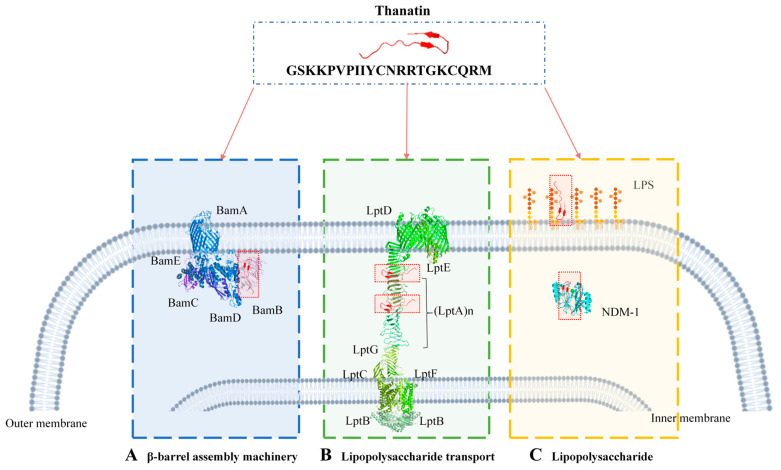
Multiple antimicrobial mechanisms of thanatin. (**A**) Thanatin targets LptA and LptD in lipopolysaccharide transport systems. (**B**) Thanatin targets BamB in a β-barrel machine. (**C**) Thanatin targets LPS. In NDM-1-producing bacteria, thanatin can bind to LPS and enter cells to bind NDM-1.

The outer membrane component biosynthesis system is widely recognized as a vulnerability in antibiotic-resistant bacteria, and thanatin effectively targets this system to exert antimicrobial effects. The initial interaction between thanatin and the bacterial membrane, encompassing binding and permeation, constitutes a crucial step in its antimicrobial mechanism. This review aims to comprehensively delineate the diverse modes of action employed by thanatin, including its interactions with LptA and LptD in lipopolysaccharide transport systems, BamB in β-barrel machines, and lipopolysaccharide (LPS) (Figure 2).

### 5.1. Targeting Lipopolysaccharide Transport

The lipopolysaccharide (LPS) transport systems play a pivotal role in protecting bacteria from the external milieu [59,60], maintaining the structural integrity of the bacterial outer membrane [61], and facilitating essential biological functions associated with LPS [62]. These functions collectively enable bacteria to thrive and adapt in diverse and harsh environments. The LPS transport systems consist of seven transport proteins, namely, LptA-LptG [63,64,65,66,67], among which LptA and LptD are specifically targeted by thanatin.

#### 5.1.1. Targeting LptA

LptA functions as the periplasmic component within the seven essential Lpt complexes (LptA to LptG), forming a stable polymerized “transmembrane bridge” structure [68]. This structure facilitates the transport of LPS across the periplasmic space to the LptDE complex embedded in the outer membrane [64,69]. Inhibition of LPS transport via binding to the periplasmic protein LptA is one mechanism through which thanatin exerts its antimicrobial effect, which is pivotal for its activity against Gram-negative bacteria [39].

Evidence implicating LptA as a target of thanatin originates from the identification of spontaneous thanatin-resistant *E. coli* (Than^R^) mutants. All isolated Than^R^ mutants, including those harboring mutations such as D31N and Q62L in LptA, demonstrated resistance to thanatin upon introduction of the mutated LptA gene into *E. coli* strains ATCC25922 or K12 MG1655 [39]. Furthermore, Moura et al., using a Bacterial Adenylate Cyclase Two-Hybrid (BACTH) assay, observed that thanatin did not inhibit the interaction between LptC and LptA^Q62L^; instead, this interaction was strengthened in the presence of thanatin [39].

NMR studies have identified complexes of thanatin with LptA from *E. coli* and *P. aeruginosa* [46], although interactions with LptA from other pathogens remain unexplored. Spectroscopic methods assessing the binding affinity of thanatin to LptA revealed a binding dissociation constant (K_d_) in the range of 12–20 nM at low molar concentrations [40]. Javadmanesh et al. investigated the interaction between thanatin and LptA from *E. coli* and *P. aeruginosa*, finding that thanatin binds to the N-terminus of LptA and disrupts the formation of LptA bridges [47]. Based on the NMR structure of the LptA-thanatin complex, Vetterli et al. determined that thanatin binds to sites within the N-terminal region of LptA that are critical for LptA oligomerization. This binding interferes with interactions essential for the assembly of the periplasmic bridge linking the inner and outer membranes, thereby impeding LPS transport [40].

The binding site of thanatin on the N-terminal domain of LptA overlaps with regions crucial for LptA oligomer formation, as well as interactions with LptC and LptD, suggesting that thanatin likely inhibits multiple protein–protein interactions (PPIs) including LptC-LptA, LptA-LptA, and LptA-LptD interactions [39,40]. These interactions are vital for the assembly of the trans-envelope Lpt bridge [70,71].

The NMR structure of the LptA_m_ and thanatin complex reveals that the N-terminal β-hairpin of thanatin (Pro7-Asn12) aligns parallel to the first β-strand (Pro35-Ser40) within the β-jellyroll of LptA_m_, facilitating close interaction evidenced by NOE contacts between these regions [40]. Beyond the thanatin β-turn (Arg13-Gly16), the C-terminal chain (Lys17-Met21) remains solvent-exposed without direct interaction with LptA_m_. Key residues such as Ile8 and Tyr10 of thanatin, along with Phe54, Ile38, and Ile86 of LptA_m_, form extensive van der Waals interactions within a hydrophobic pocket. Particularly, Tyr10 of thanatin engages in π-π stacking with Phe54 of LptA_m_. Additionally, Val6 and Pro7 of thanatin interact hydrophobically with Pro35 and Ile36 near the N-terminus of LptA_m_, influencing the orientation of the N-terminal segment of thanatin and positioning the short N-terminal helix of LptA_m_, including Asp31 [40]. Hydrogen bonds are observed between the backbone carbonyl oxygens of Ser40 and Asp41 in LptA_m_ and the amide side chain protons of Asn12 in thanatin. Thanatin residues Arg13 and Arg14 are situated near Glu39 and Asp41 of LptA_m_, respectively, facilitating potential salt bridge formation [40].

Thanatin demonstrates high-affinity binding specificity to LptA or LptA_m_, disrupting the transport of newly synthesized lipopolysaccharides (LPS) to the outer membrane, which can lead to LPS accumulation and eventual Gram-negative bacterial death [18]. Moura et al. elucidated the inhibitory mechanism whereby thanatin disrupts the interaction between LptC and LptA, both in vivo and in vitro, in a dose-dependent manner. Thanatin competitively binds and disrupts the LptC-LptA interface, effectively preventing the formation of the LptC-LptA complex. While it also interacts with LptA, its impact on the LptA-LptA interaction is less pronounced and not dose-dependent. Thus, thanatin’s primary inhibitory mechanism lies in its disruption of the LptC-LptA interaction, crucial for LPS transport in Gram-negative bacteria [39].

#### 5.1.2. Targeting LptD

The outer membrane protein LptD, characterized by its β-barrel structure, plays a critical role in the biogenesis of Gram-negative bacterial outer membranes [71,72,73]. The process of inserting lipopolysaccharides (LPS) into the outer membrane hinges upon coordinated movements within LptD’s β-barrel and β-taco domains. Thanatin functions as an antimicrobial peptide by stabilizing the β-taco domain of LptD through direct binding, thereby disrupting the transport of LPS to the outer membrane. This mechanism represents a pivotal defense strategy against microbial surfaces [74].

Vetterli et al. conducted fluorescence polarization and thermography assays to examine the binding affinity between thanatin and LptD, revealing a low nanomolar dissociation constant (K_d_) ranging from 34 to 44 nM [40]. Robinson et al., through modeling studies, identified a conserved binding site for thanatin within the periplasmic region of LptD, where it interacts specifically with the N-terminal β-strand of the β-jellyroll domain [71]. Fiorentino et al. employed natural mass spectrometry, hydrogen–deuterium exchange mass spectrometry, and molecular dynamics simulations to investigate the dynamic impact of thanatin on transport protein dynamics. Their findings indicated that thanatin binds directly to the N-terminal region of the β-taco domain, thereby hindering the influx of LPS into the outer membrane [74]. Competitive binding experiments demonstrated that both Re-LPS and thanatin can bind simultaneously to LptD/E, albeit in non-competitive manners, with LptD/E sometimes binding to Re-LPS/thanatin in a 2:1 ratio.

Thanatin exhibits differential binding characteristics with LptD across bacterial strains, penetrating the outer membrane domain of *E. coli* LptD through its β-hairpin structure, potentially obstructing the path of LPS or other substrates from LptA to LptD [47]. However, in *P. aeruginosa*, thanatin interacts exclusively with the outer regions of LptD without exerting inhibitory effects [47].

### 5.2. Targeting the β-Barrel Assembly Machinery

The β-barrel assembly machinery (Bam) is a complex assembly apparatus essential for the folding of β-barrel outer membrane proteins (OMPs) in Gram-negative bacteria, comprising BamA as the central β-barrel protein and four associated lipoproteins (BamB-BamE) [75,76]. This machinery plays a crucial role in the biogenesis of OMPs [77,78]. BamB specifically interacts with the polypeptide translocation-associated domain 3 (POTRA3) of BamA, influencing conformational changes within the Bam complex [79,80].

Vetterli et al. employed photoaffinity labeling using thanatin light probes to identify potential interaction partners of membrane proteins, followed by comprehensive mass spectrometry (MS)-based proteomic analyses. Quantitative analysis revealed specific and light-labeling-dependent enrichment of LptD, LptA, and BamB, with BamB showing lower enrichment compared to LptD and LptA [40]. However, it remains unclear whether thanatin binds to other components of the Bam complex or directly to BamB in vitro, and the precise binding mechanism between thanatin and BamB remains to be elucidated. The current literature on the interaction between thanatin and BamB is limited, underscoring the need for further investigation.

### 5.3. Targeting LPS

Lipopolysaccharide (LPS) is an essential constituent of the outer membrane in Gram-negative bacteria, serving as a primary barrier against harmful agents including antibiotics and host proteins [81,82,83]. Upon cell division or under antibiotic stress, LPS can be released from bacteria [7]. The interaction between thanatin and LPS on the outer membrane is critical for bacterial eradication and LPS neutralization [84]. Thanatin binds to lipopolysaccharides present on the bacterial membrane surface, inducing changes in outer membrane permeability and inner membrane depolarization. The binding affinity (K_d_) of thanatin for LPS ranges between 1.5 and 1.09 nM [16,48].

Direct evidence of thanatin inhibiting LPS transport was obtained through comprehensive proteomic analyses using robust mass spectrometry techniques, which identified potential interaction partners following light labeling. Indirect evidence was provided by transmission electron microscopy of *E. coli* cells treated with thanatin, revealing the accumulation of membrane-like material within cells: a typical manifestation observed when components of the Lpt complex are down-regulated [71].

Thanatin exists in LPS micelles as a dimeric structure characterized by four antiparallel β-sheets arranged in a “head-to-tail” configuration, contrasting with its monomeric form. The dimeric structure enhances thanatin’s cationic properties and exposes hydrophobic surfaces, facilitating interactions with multiple LPS molecules. This capability enables thanatin to effectively approach bacterial cells. Upon entry into the bacterial body, the LPS-thanatin complex rapidly stabilizes within 40–50 ns, with dimeric thanatin employing multiple binding modes or dynamic interactions with multiple LPS molecules [85]. Most residues of thanatin, encompassing cationic, aromatic, and nonpolar types, primarily interface with LPS micelles. The two subunits of dimeric thanatin form a complex interaction network with multiple LPS molecules through electrostatic, polar, and van der Waals contacts, concentrated primarily in the head region. Additionally, complexes involving thanatin induce structural rearrangements in LPS micelles or their binding to larger LPS aggregates. Notably, the nonpolar side chains of residues I8, I9, and M21 maintain close contact with LPS micelles. Furthermore, the side chains of cationic residues K3, K4, and R20 from one subunit of dimeric thanatin, along with residues R13′ and R14′ from the other subunit, may interact with the hydroxyl group of the sugar residue of LPS [39].

Dimeric thanatin adheres tightly to the LPS surface through two distinct binding modes. In binding mode I, residues from the N-terminal tail of one subunit and the cationic residues R13 and R14 from the central ring of the other subunit establish strong interactions with the LPS molecule. Specifically, R13 and R14 form multiple hydrogen bonds with the hydroxyl group of LPS. Additionally, residues P5′, V6′, P7′, and I8′ engage in nonpolar interactions with the sugar ring of LPS. In binding mode II, both N-terminal tails of the dimer interact actively with the LPS surface. The alkaline residues K3′ and K4′ in the N-terminal tail of dimeric thanatin create electrostatic interactions with the phosphate groups of LPS. This allows thanatin to cover the bacterial cell surface in a carpet-like manner, disrupting the integrity of the outer cell membrane, neutralizing surface charges, and ultimately causing cell agglutination to achieve its bactericidal effect [16].

Once the outer membrane is compromised, thanatin, potentially existing as a monomer, gains access to the periplasmic space. There, it interacts with the Lpt protein complex, inhibiting the translocation of LPS to the outer membrane. This disruption in LPS transport further compromises the stability and permeability of the outer membrane, leading to cell agglutination [85]. Mutant variants such as R13R14AA and Y10M12AA show reduced affinity for LPS compared to native thanatin [16]. Moura et al. introduced a mutant version of thanatin where Arg 13 and Arg 14 residues were substituted with Ala, resulting in decreased LPS binding affinity and loss of antimicrobial activity [39].

Thanatin exhibits a distinctive antimicrobial mechanism against NDM-1-producing bacteria, which carry the *bla*_NDM-1_ gene [86,87,88]. These pathogens are resistant to all β-lactam antibiotics, including carbapenems [89,90]. Thanatin exerts its potency by competitively displacing bivalent cations on the outer membrane to bind with LPS, facilitating cellular penetration and effectively eliminating NDM-1-producing bacteria [48]. Once the outer membrane is breached, thanatin accesses the cell interior, where it competitively replaces the bivalent Zn^2+^ cation of NDM-1. This disrupts the enzyme’s active site and inhibits its hydrolytic activity. This specific mechanism is unique to thanatin and not a general trait of all cationic antimicrobial peptides, including colistin [48]. Moreover, thanatin demonstrates therapeutic potential in mice infected with NDM-1-producing bacteria, significantly improving their survival rates while exhibiting minimal toxicity. Notably, even at nanomolar concentrations, thanatin can reverse resistance to carbapenem antibiotics in mice infected with NDM-1-producing *E. coli* [48].

## 6. Conclusions and Prospects

**Figure 3:**
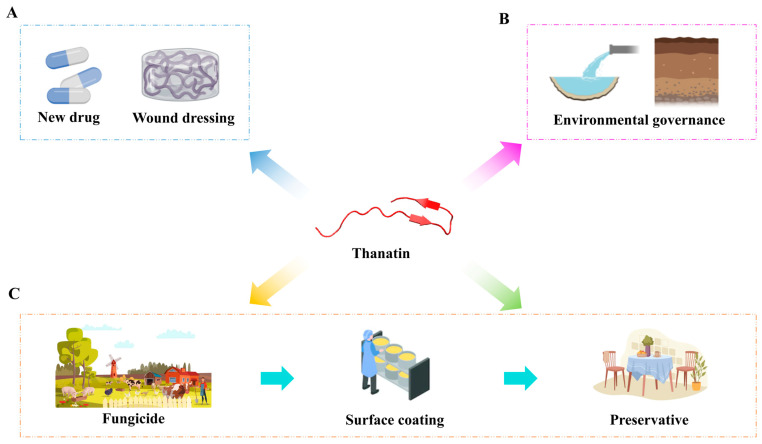
The prospects of diversified application of thanatin. (**A**) Potential clinical applications of thanatin. (**B**) Potential environmental applications of thanatin. (**C**) Potential applications of thanatin in food and agriculture.

Thanatin is recognized for its promising antimicrobial properties targeting the vulnerabilities of antibiotic-resistant bacteria, featuring broad-spectrum activity [8] and minimal toxicity [36], coupled with resistance to resistant induction [51]. Its small molecular size and well-defined structure–activity relationship make thanatin highly adaptable, suggesting significant therapeutic potential against microbial infections, notably in combating drug-resistant and multidrug-resistant strains. Considering these advantages, despite being a relatively long therapeutic peptide composed of 21 amino acids, thanatin is still regarded as a highly valuable treatment option. Moreover, thanatin holds promise for diverse applications across clinical, environmental, and food and agriculture sectors. This review underscores the prospective roles of thanatin in these domains (Figure 3). Whilst the mechanisms of action of thanatin against Gram-negative bacteria are well covered in this review, the corresponding mechanisms of action against Gram-positive bacteria and fungi not well-established. Therefore, future research should focus on the unique biological characteristics of these microorganisms to further deepen understanding of the antimicrobial and antifungal mechanisms of thanatin.

### 6.1. Clinical Applications

Amid the escalating challenge of antibiotic resistance driven by misuse, conventional antibiotics are increasingly limited in effectiveness [3]. Thanatin, with its distinct mechanism of action independent of antibiotics, demonstrates potent bactericidal activity against a wide spectrum of antibiotic-resistant bacteria [48]. This positions thanatin as a highly promising candidate for new drug research and development, offering a potential solution to the crisis of antimicrobial resistance. Moreover, due to its anti-inflammatory properties, thanatin holds promise as an antimicrobial agent in wound dressings, such as hydrogels, facilitating wound healing and preventing bacterial infections. However, it is crucial to emphasize that further research and clinical trials are essential for validating the safety and efficacy of thanatin in practical clinical applications.

### 6.2. Environmental Applications

With the mounting accumulation of pollutants in soil and water ecosystems, environmental contamination has become a critical global concern [91]. A healthier environment has the potential to prevent nearly a quarter of the global disease burden [92]. Thanatin presents a promising approach to addressing diverse environmental pollutants, particularly in sewage treatment and soil remediation. Its application can effectively mitigate pollution issues arising from bacteria and other harmful microorganisms, thereby reducing environmental pollutants and lowering the risk of antimicrobial resistance. Thanatin offers a highly sustainable and environmentally friendly strategy for tackling microbial environmental challenges, promising to play a pivotal role in safeguarding environmental integrity in the future.

### 6.3. Applications in Food and Agriculture

Due to the robust adaptability of microorganisms in ecological settings, every stage of the food production chain, from farm to table, remains susceptible to microbial contamination [93,94]. Thanatin emerges as a promising solution in agricultural contexts, serving as a potent fungicide to enhance disease resilience in both animals and plants. By reducing reliance on chemical pesticides and mitigating the risk of antibiotic resistance, it promotes sustainable agricultural practices. Additionally, thanatin can be utilized as a surface coating on processing equipment to effectively inhibit biofilm formation. Furthermore, its application as a preservative during food processing extends shelf life, preserves food quality, and diminishes dependence on chemical additives throughout storage, transportation, and marketing phases.

## Figures and Tables

**Table 1:** Antimicrobial activity of thanatin, S-thanatin, and A-thanatin against Gram-negative bacteria.

Microorganism	MIC			Reference
	Thanatin	A-Thanatin	S-Thanatin	
Gram-negative bacteria				
*Escherichia coli* D22	0.6 µM	1.2 µM	-	[8]
*E. coli* 1106	1.2 µM	1.2 µM	-	[8]
*E. coli* ATCC 25922	1.6 µM	6.4 µM	1.6 µM	[24,44,48]
*E. coli* ATCC 35218	0.4 µM	-	-	[48]
*E. coli* BL21	0.5 µM	-	-	[19]
*E. coli* E79466	-	-	5 µM	[43]
*E. coli* O157:H7	2.5 µM	-	-	[47]
*E. coli* k99	5 µM	-	-	[47]
MDR-EC XJ74283	-	3.2 µM	-	[44]
MDR-EC JT11092	-	0.8 µM	-	[44]
ESBL-EC ATCC35218	-	1.6 µM	-	[44]
ESBL-EC HN10318	-	0.8 µM	-	[44]
ESBL-EC JT11074	-	0.8 µM	-	[44]
ESBL-EC HN10322	-	0.8 µM	-	[44]
ESBL-EC SX49657	-	1.6 µM	-	[44]
ESBL-EC SX49660	-	1.6 µM	-	[44]
NDM-1 *E. coli* XJ141015	0.8 µM	-	-	[48]
NDM-1 *E. coli* XJ141026	0.8 µM	-	-	[48]
NDM-1 *E. coli* XJ141047	0.8 µM	-	-	[48]
*Salmonella typhimurium*	1.2 µM	1.2 µM	-	[8]
*Salmonella enterica* ATCC 14028	1 µM	-	-	[19]
*Salmonella paratyphi C*	7.692 µM	-	-	[31]
*Klebsiella pneumoniae*	1.2 µM	1.2 µM		[8]
*K. pneumoniae* ATCC 700603	3.2 µM	-	1.6 µM	[24]
*K. pneumoniae* ATCC 13883	1 µM	-	1.6 µM	[1,19]
NDM-1 *K. pneumoniae* XJ155017	3.2 µM	-	-	[48]
NDM-1 *K. pneumoniae* XJ155018	3.2 µM	-	-	[48]
NDM-1 *K. pneumoniae* XJ155019	3.2 µM	-	-	[48]
NDM-1 *K. pneumoniae* XJ155020	3.2 µM	-	-	[48]
ESBL-KP XJ75297	-	1.6 µM	-	[44]
ESBL-KP HN10349	-	0.8 µM	-	[44]
ESBL-KP HN10500	-	0.8 µM	-	[44]
*Klebsiella ornithinolytica* ATCC 31898	-	-	3.2 µM	[23]
*Klebsiella oxytoca* ATCC 43086	-	-	3.2 µM	[23]
*Enterobacter cloacae*	2.5 µM	2.5 µM	-	[8]
*E. cloacae* ATCC 13047	-	-	3.2 µM	[23]
*Erwinia carotovora*	20 µM	20 µM	-	[8]
*Pseudomonas aeruginosa*	40 µM	5 µM	-	[8]
*P. aeruginosa* ATCC 27853	1 µM	-	12.8 µM	[1,19]
MDR-PA XJ75315	-	6.4 µM	-	[44]
*Enterobacter aerogenes* ATCC 49701	-	-	1.6 µM	[23]
*Citrobacter freundii*	6 µM	-	-	[17]
*Erwinia amylovora*	6.248 µM	-	-	[49]
*Shigella dysenteriae*	15.384 µM	-	-	[31]

Note: - No data.

**Table 2:** Antimicrobial activity of thanatin, S-thanatin, and A-thanatin against Gram-positive bacteria.

Microorganism	MIC			Reference
	Thanatin	A-Thanatin	S-Thanatin	
Gram-positive bacteria				
*Aerococcus viridans*	1.2 µM	1.2 µM	-	[8]
*Micrococcus luteus*	2.5 µM	2.5 µM	-	[8]
*Bacillus megaterium*	5 µM	1.2 µM	-	[8]
*Bacillus subtilis*	2 µM	5 µM	-	[8,19]
*B. subtilis* ATCC 21332	1.6 µM	-	3.2 µM	[15]
*Staphylococcus aureus*	-	40 µM	-	[8]
*S. aureus* ATCC 25923	1 µM	-	-	[19]
*S. aureus* ATCC 29213	-	102.4 µM	-	[44]
*S. haemolyticus* XJ31196	1.6 µM	-	-	[46]
*S. haemolyticus* XJ31245	0.8 µM	-	-	[46]
*S. haemolyticus* SX92421	3.2 µM	-	-	[46]
*S. haemolyticus* SX92464	1.6 µM	-	-	[46]
*S.hominis* XJ31287	0.8 µM	-	-	[46]
*S.hominis* XJ31303	3.2 µM	-	-	[46]
*S.hominis* SX92357	1.6 µM	-	-	[46]
*S.hominis* SX92433	0.8 µM	-	-	[46]
MRSA ATCC 33591	3.844 µM	-	-	[31]
MRSA XJ75302	25.6 µM	>102.4 µM	-	[44,46]
MRSE XJ75284	1.6 µM	6.4 µM	-	[44,46]
MRSE XJ31106	0.8 µM	-	-	[46]
MRSE XJ31204	1.6 µM	-	-	[46]
MRSE XJ31276	1.6 µM	-	-	[46]
MRSE XJ31318	3.2 µM	-	-	[46]
MRSE SX70535	0.8 µM	-	-	[46]
MRSE SX70582	1.6 µM	-	-	[46]
MRSE SX70810	1.6 µM	-	-	[46]
MRSE SX70892	3.2 µM	-	-	[46]
MRSE SX70893	1.6 µM	-	-	[46]
*Pediococcus acidolactici*	40 µM	40 µM	-	[8]
*Enterococcus faecalis* ATCC 29212	0.5 µM	-	25.6 µM	[1,19]
*Streptococcus pyogenes* ATCC 19615	0.5 µM	-	-	[19]
*Listeria monocytegenes*	30.768 µM	-	-	[31]

Note: - No data.

## Data Availability

No new data were created or analyzed in this study.
